# Clinician compliance to recommendations regarding the risk of suicidality with selective serotonin reuptake inhibitors in the treatment of children and adolescents

**DOI:** 10.1007/s00787-019-01435-0

**Published:** 2019-11-07

**Authors:** Johanne Østerby Sørensen, Annette Rasmussen, Troels Roesbjerg, Anne Katrine Pagsberg

**Affiliations:** 1 Department of Clinical Medicine, Faculty of Health and Medical Sciences and Child and Adolescent Mental Health Center, Mental Health Services, Capital Region of Denmark and University of Copenhagen, Kildegaardsvej 28, Opgang 3A, 1 sal, 2900 Hellerup, Denmark; 2 Mental Health Services, Capital Region of Denmark, Kristineberg 3, 2100 Copenhagen Ø, Denmark

**Keywords:** Selective serotonin reuptake inhibitors, Children and adolescents, Adverse effects, Suicidality, Guideline compliance

## Abstract

Meta-analyses of randomized-controlled trials have established a heightened risk of suicidality for children and adolescents treated with selective serotonin reuptake inhibitors (SSRIs). The present study examined to what extent daily clinical practice complied with specific recommendations regarding the risk of suicidality when treating children and adolescents with SSRIs. All in- and outpatients aged 0–17 years at the Child and Adolescent Mental Health Services, Capital Region of Denmark with a prescription for SSRI on January 1st, 2016 were identified. Data were obtained for *n* = 365 patients regarding the level of clinician compliance to deliver pre-consent information about adverse effects, monitor for suicidality, and provide non-pharmacological interventions. 81.7% (*n* = 298) of patients received pre-consent information about adverse effects. 67.7% (*n* = 247) were monitored for suicidality within 6 weeks after SSRI initiation. Children (0–13 years) were less likely to be monitored for suicidality compared to adolescents (14–17 years) (49.6% vs. 77.5%, *p* < 0.001). Patients with depression as indication for SSRI treatment were more likely to be monitored for suicidality than patients with other indications (OR = 3.4, *p* = 0,002) and more likely to receive information specifically about suicidality (34.7% vs. 19.7%, *p* = 0.002). Respectively, 89.3% (*n* = 326) and 93.4% (*n* = 341) of all SSRI-treated patients were treated with non-pharmacological interventions prior to and in parallel with SSRI treatment. For the majority of cases, treatment of children and adolescents with SSRI complied with recommendations from clinical guidelines. However, patients of younger age and/or with indications for SSRIs other than depression were less likely to be managed according to recommendations.

## Introduction

In recent years, the use of antidepressant drugs among children and adolescents has increased in western countries [1, 2]. Several meta-analyses of data from randomized-controlled trials (RCT’s) have found an increased risk of suicidality for children and adolescents treated with antidepressants [3–7]. In 2004, The American Food and Drug Administration (FDA) directed that the labelling of all antidepressants should include a boxed warning of an increased risk of suicidality for children and adolescents [8], and in 2007, this was expanded to include young adults (ages 18–24 years) [9]. In 2005, The European Medicines Agency issued a warning about the use of antidepressants for children and adolescents [10]. Likewise, clinical guidelines covering the use of antidepressants for children and adolescents have taken the risk of suicidality into consideration [11–16]. The guidelines generally recommend non-pharmacological interventions as first-line treatment for children and adolescents with depression, anxiety, and OCD, and if antidepressants are necessary, they should be used as a supplement to boost non-pharmacological interventions. Most guidelines recommend careful monitoring for suicidality as an adverse effect after initiation of antidepressant treatment. However, the guidelines differ regarding what time period and for what diagnostic indications for antidepressants, the risk of suicidality should be considered. These differences reflect that for the indication of major depressive disorder, suicidality as an adverse effect has been well described, but the risk of suicidality is not as clearly established when antidepressants are given on other diagnostic indications [6]. Neither has the present literature fully described in what time period during treatment, antidepressant-related suicidality might occur [17–19]. The guidelines have no specific recommendations for monitoring and information about adverse effects during dosage changes. In many of the RCTs, patients with high risk of suicide and many co-morbid disorders were excluded [3, 20], thus complicating extrapolation to a clinical population, why observational studies are necessary to describe a more representative population and reflect clinical practice. The present study aims to determine whether the antidepressant or in particular SSRI treatment of children and adolescents in daily clinical practice complies with specific national recommendations regarding suicidality from The Danish Health Authority. The hypothesis of the study is that SSRI treatment of children and adolescents is in concordance with clinical recommendations, thus:

Clinicians give information about suicidality as an adverse effect of SSRIs to patients and their parents.
Patients are monitored for suicidality during the first 6 weeks of treatment (or longer if needed).
(a) Non-pharmacological interventions are first-line treatment before SSRI treatment is initiated.
  (b) SSRIs are used as a supplement to boost non-pharmacological interventions.

The hypothesis is based on recommendations from The Danish Health Authority clinical guidelines issued in 2013. Previous guidelines were issued by the Danish Health Authority in 2012 and 2007. The guidelines issued in 2012 and 2007 were similar in terms of recommendation of non-pharmacological interventions before and in parallel with SSRI treatment and the management of SSRI-related suicidality. However, the guidelines from 2007 were less explicit on the management of suicidality as an adverse effect in comparison to the guidelines from 2012 and 2013 [21].

## Methods

### Design

A cross-sectional study with follow-back and follow-up was conducted on January 1st 2016 based on data extracted from electronic prescription software (PLISS) used for all prescriptions at Child and Adolescent Mental Health Services (CAMHS), Capital Region, Denmark. All patients have a unique id number which was used to extract data from the digitalized medical record (OPUS) and the electronic medicine monitoring system (EPM).

### Study population

#### Inclusion criteria

All in- or outpatients aged 0–17 years with a recorded prescription for an SSRI classified within ATC-group N06AB undergoing treatment at the CAMHS. CAMHS is a public mental health center which holds the main responsibility for the hospital-based treatment in the Capital Region of Denmark, encompassing 8752 unique in- and outpatients in 2015. The Capital Region is the most populous region of Denmark with a population of 359441 children (aged 0–17) in 2015 [22]. In Denmark, free health care is available to all citizens and financed via income taxes.

#### Exclusion criteria

Patients aged 18 years and above on the date of SSRI initiation and patients who were not undergoing SSRI treatment on January 1st 2016 according to the medical record were excluded. Cases where SSRI treatment was not initiated in CAMHS (i.e., SSRI treatment was initiated in a private child- and adolescent psychiatric practice, or another practitioner, not part of CAMHS) were excluded, because precise information about the initiation and management of medical treatment was not available in the CAMHS medical records. For the same reason, cases in which it was impossible to determine who initiated the SSRI treatment were excluded. Cases with another recorded prescription for SSRI prior to the SSRI which was prescribed on January 1st 2016 were excluded.

#### Case extraction

Patients with a prescription for an SSRI were identified by one researcher (TR) in June 2016. Two researchers (JØS and AR) searched the medical records and the medicine monitoring system for cases. For each case, the medical record was searched manually for relevant data from the first entry in the course of treatment that included SSRI prescription until the date of SSRI initiation (termed: the period prior to SSRI initiation), and from SSRI initiation date until 6 months after treatment initiation or until the patient was no longer a patient in CAMHS (termed: the period after SSRI initiation). The date of SSRI initiation ranged from year 2009 to 2015.

### Data collection

For each patient, the following data were extracted:

1. Date of study entry and end of follow-up
2. Gender
3. Age on SSRI initiation date. Patients were grouped as children (aged 0–13 years) or adolescents (aged 14–17 years)
4. Patient status (in- or outpatient) on initiation date
5. Data on SSRI use: initiation date, type of drug, indication, initiation dose, maximum dose, and maintenance dose. Data on the date of dose changes were not available.
6. Medication status prior to, on the day of, and after SSRI initiation
7. Psychiatric diagnoses according to ICD-10 [23]. Since diagnoses could be changed during the study period, diagnoses from an entry immediately before or on the initiation date were extracted. Tentative diagnoses were included if a firm diagnosis was not yet established.
8. Suicidality (according to C-CASA) [24] prior to SSRI initiation.

### Outcome variables

#### Pre-consent information about suicidality as an adverse effect of SSRI

The medical records were searched for entries regarding information from healthcare providers to the patient and/or their parent(s) concerning SSRI adverse effects on the date of or just prior to SSRI initiation. Information from the healthcare providers was registered as general information about adverse effects or specifically about suicidality as an adverse effect and to whom the information was given (parents, patient, or both) according to the medical record.

#### Monitoring for suicidality

In the period after SSRI initiation, the medical records were searched for screenings for suicidality conducted by the healthcare providers, suicidality assessments, or entries such as: *“(no) suicidal ideations”* or *“(not) suicidal”.* Monitoring face-to-face, by telephone and whether a side-effect rating scale was used to aid the evaluation of suicidality was registered. Phrases indicating a general assessment of adverse effects such as *“no adverse effects”* were not registered as valid monitoring for suicidality.

#### Non-pharmacological interventions

Non-pharmacological interventions were grouped in four main categories: psychotherapy, psychoeducation, supportive consultations, and reducing psychosocial stress factors. The categories were defined beforehand and adapted from The Danish Association for Child and Adolescent Psychiatry’s clinical guidelines for management and treatment of depression and OCD [25, 26]. A category termed “Other” was used if the non-pharmacological intervention did not match one of the main categories. Every recorded non-pharmacological intervention before and after SSRI initiation was registered in the period prior to SSRI initiation and in the period after SSRI initiation. If the same non-pharmacological intervention appeared more than once during the same period, the intervention was counted only once. If an intervention was delivered outside CAMHS, the intervention was registered separately. However, if the same specific intervention was also performed by the CAMHS healthcare providers, only the CAMHS intervention was registered.

### Statistical analysis

Statistics were performed using IBM Corp, IBM SPSS Statistics for Windows Version 22. The Mann–Whitney *U* test was applied to test for significant age difference between males and females. The Pearson Chi-square test and Fisher’s exact test were used to evaluate the outcomes of treatment, monitoring and information about adverse effects according to age, gender, admission status, indication for SSRI, and suicidality prior to SSRI initiation. To analyse monitoring for suicidality according to age, gender, admission status, indication, and previous suicidality, a logistic regression analysis was performed with monitoring as the dependent variable and age group, gender, admission status, indication (depression or no depression), and previous suicidality as predictor variables.

### Ethical aspects

The study was approved by the Danish Data Protection Agency (Journal no.: 2012-58-0004) and the Danish Patient Safety Authority (Journal no. 3-3013-1554/1 + 2/). Informed consent from patients and caretakers was not demanded. Approval from the Ethics Committee was not required according to Danish Law.

## Results

**Fig. 1:**
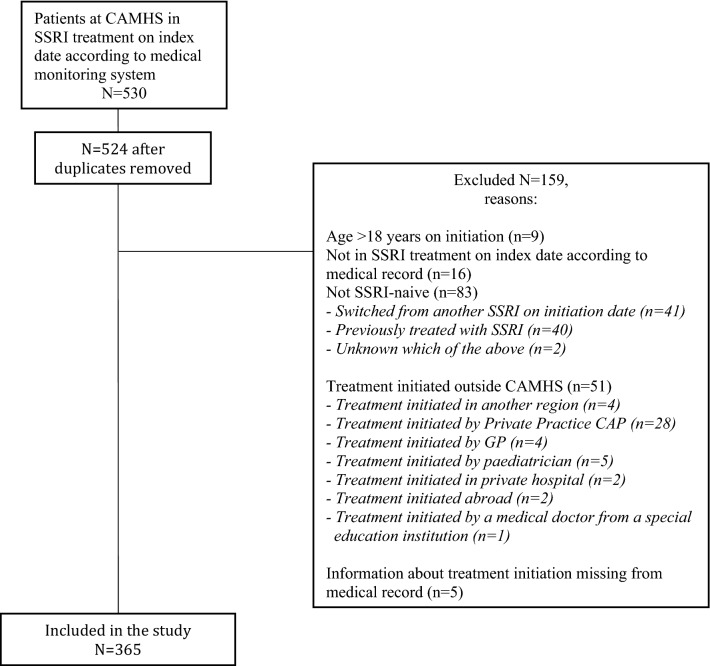
Flowchart of inclusion of patients. 530 patients were identified as SSRI users on index date. 159 patients were excluded according to exclusion criteria. Consequently, 365 patients were eligible for study inclusion

**Fig. 2:**
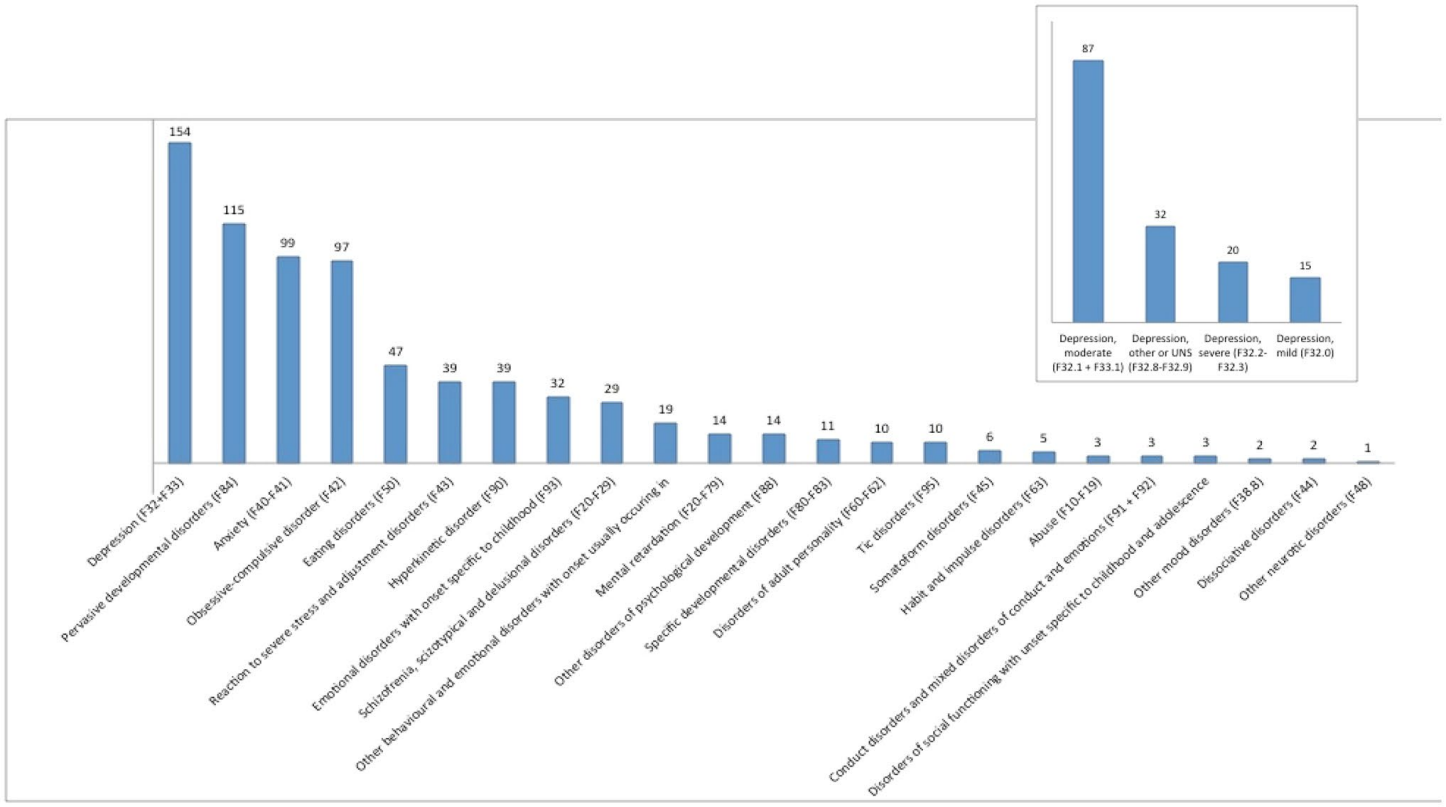
Psychiatric diagnoses in the study population. Most patients, 71.5% (*n* = 261), had more than one diagnosis on initiation accordingly; the number of diagnoses are higher than the number of patients included in the study. If a patient had more than one diagnosis from the same diagnosis group, it was counted only once in this figure. Depression, pervasive developmental disorders, anxiety, and OCD were the most abundant diagnoses. The insert square depicts the types of depression

In total, 5410 patients were associated with CAMHS on January 1st 2016. Out of 530 patients identified as SSRI users on initiation date, 365 patients were eligible for study inclusion. Figure 1 shows the inclusion of patients in the study and Table 1 summarizes patient characteristics. The majority (64.1%, *n* = 234) was female. Mean [SD] age was 14.5 years [2,04], ranging from 7.7 to 17.9 years, and was significantly higher for females than for males, (14.8 years [1.8] vs. 13.6 years [2], *p* < 0.001). More patients were adolescents (aged 14–17) compared to children (aged 0–13) (64.7%, *n* = 236 vs. 35.5%, *n* = 129, *p* < 0.001). Most patients were outpatients (77.8%, *n* = 284, *p* < 0.001). Sertraline was the most commonly used SSRI, (75.7%, *n* = 280), followed by fluoxetine (23.0%, *n* = 84). Only 0.3% (*n* = 1) of patients received Citalopram. The most common indication for SSRI treatment was depression (40.3%, *n* = 147), followed by anxiety, (29.6%, *n* = 108) and OCD (23.8%, *n* = 87). The majority (67.9%, *n* = 253) of patients had displayed suicidality prior to SSRI initiation. Adolescents more frequently displayed pre-SSRI suicidality than children (75.2%, *n* = 178 vs. 58.1% *n* = 75, *p* = 0.001) and females more frequently than males (75.2%, *n* = 176 vs. 58.8%, *n* = 77, *p* = 0.003) Fig. 2.

**Table 1:** Characteristics of study population

	No. of patients (%)	Children (%)	Adolescents (%)	*p*^a^	
Adolescents (14–17 yr)	236 (64.7%)				
Children (7–13 yr)	129 (35.3%)			< 0.001	
Gender					
Female	234 (64.1%)	59 (45.7%)	175 (25.8%)		
Male	131 (35.9%)	70 (54.3%)	61 (74.2%)	< 0.001	
Patient status at SSRI initiation					
Outpatient	284 (77.8%)	92 (71.3%)	192(81.4%)		
Inpatient	81 (22.2%)	37 (28.7%)	44(18.6%)		0.35
Drug					
Sertraline	280 (76.7%)	113 (87.6%)	166 (70.3%)		
Fluoxetine	84 (23.0%)	16 (12.4%)	69 (29.2%)		
Citalopram	1 (0.3%)	0	1 (0.4%)	0.001	
Indication for SSRI					
Depression	147 (40.3%)	31 (24.0%)	116 (49.2%)		
Anxiety	108 (29.6%)	39 (30.2%)	69 (29.2%)		
OCD	87 (23.8%)	51 (39.6%)	36 (15.3%)		
Depression and anxiety	9 (2.5%)	2 (1.6%)	7 (3.0%)		
Anxiety and OCD	6 (1.6%)	2 (1.6%)	4 (1.7%)		
Depression and OCD	3 (0.8%)	2 (1.6%)	1 (0.4%)		
Depression, anxiety, and OCD	2 (0.5%)	1 (0.8%)	1 (0.4%)		
“Nerve medicine”	2 (0.5%)	1 (0.8%)	1 (0.4%)		
PTSD and anxiety	1 (0.3%)	0	1 (0.4%)	< 0.001	
Number of diagnoses					
1	104 (28.5%)	37 (28.7%)	67 (28.4%)		
2	142 (38.9%)	46 (35.7%)	96 (40.7%)		
3	90 (24.7%)	34 (26.4%)	56 (23.7%)		
4	24 (6.6%)	10 (7.8%)	14 (23.7%)		
5	5 (1.4%)	2 (1.6%)	3 (1.3%)		
Previous suicidality	253 (67.9%)	75 (58.1%)	178 (75.4%)	0.001	
Other drugs at SSRI initiation					
No	218 (59.7%)	69 (53.5%)	149 (63.1%)		
Yes	147 (40.3%)	60 (46.5%)	87 (36.9%)	0.075	
Other drugs at SSRI initiation					
Melatonin	93 (25.5%)				
Quetiapine	19 (5.2%)				
Olanzapine	16 (4.4%)				
Chlorprothixene	13 (3.6%)				
Methylphendiate	13 (3.6%)				
Aripiprazole	10 (2.7%)				
Oxazepam	9 (2.5%)				
Risperidone	9 (2.5%)				
Atomoxetine	4 (1.1%)				
Lisdexamphetamine	2 (0.5%)				
Valproate	2 (0.5%)				
Lamotrigine	2 (0.5%)				
Clonidine	1 (0.3%)				

		Range	Mean (SD)	*p*^b^
Age, all (years)		[7.7–17.9]	14.5 (2.04)	
Age, females (years)		[7.7–17.9]	14.8 (1.8)	
Age, males (years)		[9.3–17.6]	13.6 (2.2)	≤ 0.001

Doses in mg.			
	Start dose (Mean)	Maintenance dose (Mean)	Max dose (Mean)
Sertraline	12.5–50 (27.3)	25–200 (105.3)	25–200 (110.0)
Fluoxetine	10–20 (10.9)	10–40 (25.4)	10–40 (26.7)
Citalopram	10	10	20

*SSRI* selective serotonin reuptake inhibitor

^a^Comparison of age groups. Pearsons Chi-square test was used to test for significance

^b^Comparison of mean age by sex. Mann–Whitney *U* test was used to test for significance

The date of SSRI initiation ranged from 14.04.2009 to 30.12.2015. 16.7% (*N* = 61) of patients started SSRI prior to the issue of the 2013 version of the guidelines from the Danish Health Authority.

### Information about suicidality as an adverse effect of SSRI

Data on information given to the patient or parent(s) about suicidality as an adverse effect of SSRI is shown in Table 2. Most patients (81.7%, *n* = 298) had an entry in the medical record concerning information given from the healthcare providers to either the patient or their parent(s) about SSRI adverse effects. In 21.4% (*n* = 78) of cases, the medical record had documentation that patients were specifically informed about suicidality as an adverse effect; 44.1% (*n* = 161) of patients were informed generally about adverse effects; and 34.5% (*n* = 126) of patients had no entry about adverse effect information. For the parents of the patients, 24.4% (*n* = 89) were specifically informed about suicidality as an adverse effect of SSRI; 52.1% (*n* = 190) were informed generally about adverse effects; and 23.6% (*n* = 86) of patients had no documentation for adverse effect information given to the parent(s). For 25.8% (*n* = 94) of the patients, either the patient or the parent(s) was informed specifically about suicidality as an adverse effect of SSRI.

**Table 2:** Clinician information to patient/parent about suicidality as an AE to SSRI

	No. of cases %	
Patient information		
Specifically about suicidality	78	21.40%
Generally about AEs	161	44.10%
Not informed	126	34.50%
Parent information		
Specifically about suicidality	89	24.40%
Generally about AEs	190	52.10%
Not informed	86	23.60%
Either parent or patient informed	298	81,7%
Either informed specifically about suicidality	94	25.80%
Both informed specifically about suicidality	73	20.00%
Both informed generally about AEs	147	40.30%

*SSRI* selective serotonin reuptake inhibitors, *AE* adverse effect

Patients with depression as indication for SSRI treatment were more likely to receive information specifically about risk of suicidality compared to patients with other diagnostic indications (34.7%, *n* = 51 vs. 19.7%, *n* = 43, *p* = 0.001).

There was no difference between the level of information given to the patient or their parent(s) according to the patient admission status. However, outpatients or their parent(s) were more likely to receive information specifically about suicidality as an adverse effect of SSRIs than inpatients or their parent(s) (28.9%, *n* = 82 vs. 14.8%, *n* = 12, *p* = 0.007).

Patients who had displayed suicidality prior to SSRI initiation or their parent(s) were more likely to receive information from the clinicians regarding adverse effects than patients with no previous suicidality (86.2%, *n* = 218 vs. 71.4%, *n* = 80, *p* = 0.001). Likewise, clinicians were more likely to inform specifically about the risk of suicidality to patients or the parent(s) of patients with previous suicidality (28.9%, *n* = 73, vs. 18.8%, *n* = 21, *p* = 0.042).

Patients or the parent(s) of patients who were initiated on SSRIs before the issue of the 2013 version of the guidelines from the Danish Health Authority were less likely to receive information from the clinicians about adverse effects (68.9%, *n* = 42 vs. 84.2%, *n* = 256, *p* = 0.005), but there was no statistically significant difference (*p* = 0.131) between the level of information specifically about the risk of suicidality before and after the issue of the 2013 version of the guidelines.

There was no significant difference between the level of information given to the patient or their parent(s) according to age group (*p* = 1.57); gender (*p* = 2.64) or indication (*p* = 0.368) and no significant difference between information specifically about suicidality given to the patient or their parent(s) according to age group (*p* = 0.134) or gender (*p* = 0.263).

### Monitoring for suicidality

Table 3 shows that the majority (83.3%, *n* = 304) of patients were monitored for suicidality in the period after SSRI initiation. Within the first 4 and 6 weeks after SSRI initiation, respectively, 59.7% (*n* = 218) and 67.7% (*n* = 247) of the patients were monitored for suicidality. When SSRIs was initiated, 51.0% (*n* = 186) of patients were monitored for suicidality.

**Table 3:** Monitoring for suicidality after SSRI initiation

	No. of patients	%	*p*
Not monitored	61	16.70%	
Monitored	304	83.30%	
Children	90	69.80%	
Adolescents	214	90.70%	0.000
Monitored first 4 weeks	218	59.70%	
Children	56	43.40%	
Adolescents	162	68.60%	0.000
Monitored first 6 weeks	247	67.70%	
Children	64	49.60%	
Adolescents	183	77.50%	0.000
Method for monitoring			
Face-to-face	269	88.5%^a^	
Telephone	4	1.3%^a^	
Face-to-face and telephone	27	8.9%^a^	
Unknown	4	1.3%^a^	
Use of side-effect rating scale^b^	85	23.3%^a^	
Monitored at SSRI initiation	186	51.00%	
Children	46	35.70%	
Adolescents	140	59.30%	0.000
Monitored with side-effect rating scale at SSRI initiation	12	6.4%^c^	

	Mean	SD	Range
Days monitored	120.1	52.57	[8–184]
No. of monitoring events	6.62	8.092	[1–63]
Frequency of monitoring by days	0.0559	0.0536	[0.01–0.36]

*SSRI* selective serotonin reuptake inhibitor

^a^Percentage of patients monitored

^b^UKU side-effect rating scale or unspecified side-effect rating scale

^c^Percentage of patients monitored at SSRI initiation

Pearsons Chi-square test was used to test for significance

Children were less likely to be monitored for suicidality after SSRI initiation than adolescents (69.8%, *n* = 90 vs. 90.7%, *n* = 214, *p* < 0.001) and this finding was consistent within the first four weeks (43.4%, *n* = 56 vs. 68.6%, *n* = 162, *p* < 0.001), and the first 6 weeks (49.6%, *n* = 64 vs. 77.5, *n* = 183, *p* < 0.001). Inpatients were more likely to be monitored for suicidality than outpatients (95.1%, *n* = 77 vs. 79.9%, *n* = 227, *p* = 0.001), within 4 weeks (79.0%, *n* = 64 vs. 54.2%, *n* = 154, *p* < 0.001) and within 6 weeks (85.2%, *n* = 69 vs. 62.7%, *n* = 178, *p* < 0.001). Patients with an indication of depression were more likely to be monitored for suicidality compared to patients with other indication diagnoses (93.9%, *n* = 138 vs. 76.1%, *n* = 166, *p* < 0.001). Patients with an indication of OCD were less likely to be monitored for suicidality than patients with other diagnostic indications (71.3%, *n* = 62 vs. 87.1%, *n* = 242, *p* = 0.001), but this relationship was not significant when stratified for age (children: *p* = 0.081, adolescents: *p* = 0.347). Patients with previous suicidality were more likely to be monitored compared to patients with no previous suicidality in the period after SSRI initiation (67.9%, *n* = 76 vs. 90.1%, *n* = 228, *p* < 0.001), within the first 4 weeks after SSRI initiation (38.4%, *n* = 43 vs. 69.2%, *N* = 175, *p* < 0.001) and the first 6 weeks (47.3%, *n* = 53 vs. 76.7%, *n* = 194, *p* < 0.001). Patients where SSRIs were initiated before the issue of the 2013 guidelines from the Danish Health Authority were less likely to be monitored (63.9%, *n* = 39 vs. 87.2%, *n* = 265, *p* < 0.001) within the first 4 weeks (36.1%, *n* = 22 vs. 64.5%, *n* = 196, *p* < 0.001) and the first 6 weeks (44.3%, *n* = 27 vs. 72.4%, *n* = 220, *p* < 0.001).

Logistic regression analyses were performed with, respectively, any monitoring during the observation period; monitoring within 4 weeks; and monitoring within 6 weeks after SSRI initiation as the dependent variable and age; gender; admission status; indication and previous suicidality as predictor variables. Table 4 gives the odds ratios (ORs) and the probability values for each of the predictor values. This shows that both increasing age, inpatient status, an indicating diagnosis of depression for SSRI, and previous suicidality increase the probability of monitoring for suicidality after SSRI initiation.

**Table 4:** Logistic regression of monitoring for suicidality after SSRI initiation

	OR (CI)	*p*
Model 1: Any monitoring		
Gender (male)	0.973 (0.518–1.829)	0.933
Increasing age	1.315 (1.131–1.529)	0.000
Indication depression^*^	2.501 (2.501–1.115)	0.026
Previous suicidality	2.429 (1.300–4.538)	0.005
Admission status (inpatient)	5.073 (1.678–15.334)	0.004
Model 2: Monitoring within 4 weeks		
Gender (male)	0.859 (0.523–1.412)	0.549
Increasing age	1.207 (1.067–1.365)	0.003
Indication depression^*^	2.045 (1.216–3.441)	0.007
Previous suicidality	2.226 (1.341–3.695)	0.002
Admission status (inpatient)	3.227 (1.709–6.093)	0.000
Model 3: Monitoring within 6 weeks		
Gender (male)	0.812 (0.486–1.357)	0.427
Increasing age	1.231 (1.085–1.397)	0.001
Indication depression^*^	2.271 (1.285–4.012)	0.005
Previous suicidality	2.175 (1.297–3.649)	0.003
Admission status (inpatient)	3.537 (1.732–7.2204)	0.001

A dummy variable for indication was created. Indication was either “depression” or “other”

### Non-pharmacological interventions

Before SSRI treatment was initiated, 89.3% (*n* = 326) of patients were treated with a non-pharmacological intervention. 57.3% (*n* = 209) of patients were treated with psychotherapy, 38.6% (*n* = 141) were treated with supportive consultations, and 27.9% (*n* = 102) were treated with psychoeducation. Almost all inpatients (97.5%, *n* = 79) received non-pharmacological interventions prior to SSRI initiation, whereas a significantly lower proportion of outpatients were treated with non-pharmacological interventions prior to SSRIs (87.9%, *p* = 0.004). There was no difference between the extent of non-pharmacological interventions prior to SSRI initiation according to age group (*p* = 111), gender (*p* = 484), indication for SSRIs (*p* = 0.388), previous suicidality (*p* = 0.722), or the SSRI initiation date (*p* = 0.363). 20.3% (*n* = 4) of the patients with a diagnosis of severe depression (*n* = 20) received no non-pharmacological intervention prior to SSRI initiation, but a diagnosis of severe depression and no prior non-pharmacological interventions (*p* = 0.103) were not related.

After SSRI initiation, 93.4% (*n* = 341) of patients were treated with a non-pharmacological intervention in parallel with SSRIs. 60.3% (*n* = 220) of patients were treated with psychotherapy, 46.8% (*n* = 171) were treated with supportive consultations, and 79.7% (*n* = 126) were treated with psychoeducation. All inpatients (100%, n = 81) received non-pharmacological interventions, whereas 91.5% (n = 260) of outpatients received non-pharmacological interventions in parallel with SSRIs (p = 0.007). Patients who were initiated on SSRIs before the issue of the 2013 version of the guidelines from the Danish Health Authority were less likely to receive non-pharmacological treatment in parallel with SSRIs (86.9%, n = 53 vs. 94.7%, n = 288, p = 0.024). There was no difference between the extent of non-pharmacological interventions after SSRI initiation according to age group: (p = 0.276), gender: (p = 0.513), indication (p = 0.401), and previous suicidality (p = 0.096).

To account for misclassification of psychotherapy for supportive consultations, the number of patients treated with either/or was calculated. Thus, the percentage of patients who were treated with psychotherapy or supportive consultations was 73.7% (*n* = 269) prior to and 79.7% (*n* = 291) in parallel with SSRI treatment (Table 5).

**Table 5:** Non-pharmacological interventions

	No. of patients	%
Before SSRI treatment		
Any	326	89.30%
Psychotherapy	209	57.30%
Supportive consultations	141	38.60%
Psychoeducation	102	27.90%
Psychotherapy or supportive consultations	269	73.70%
Psychotherapy or psychoeducation	234	64.10%
Psychotherapy or supportive consultations or psychoeducation	280	76.70%
After SSRI initiation		
Any	341	93.40%
Psychotherapy	220	60.30%
Supportive consultations	171	46.80%
Psychoeducation	126	34.50%
Psychotherapy or supportive consultations	291	79.70%
Psychotherapy or psychoeducation	261	71.50%
Psychotherapy or supportive consultations or psychoeducation	300	82.20%

*SSRI* selective serotonin reuptake inhibitor

## Discussion

In this study of SSRI-treated children and adolescents in a large Danish hospital setting, the clinical guidelines regarding information to patients or their parents, monitoring for suicidality, and use of non-pharmacological interventions were followed in the majority of cases. Yet, some patients were not managed according to guidelines and the clinical management of SSRI-related suicidality differed depending on patient age, admission status, indication for SSRI treatment, date of SSRI initiation, and whether the patient had displayed suicidality prior to SSRI initiation.

### Pre-consent information about suicidality as an adverse effect of SSRI

The majority of patients or their parent(s) were informed about adverse effects of SSRIs before treatment initiation. However, less than a quarter of the parents were informed specifically about suicidality. This finding seems alarming and violates the clinical recommendation that parent(s) should be instructed in how to support the monitoring for suicidality [11]. However, because of the study design, information is limited on what is exactly covered in the term “general information” about adverse effects. Presumably, general information given about adverse effects covers suicidality. In this study, for the majority of cases, the parent(s) were informed generally about adverse effects. Accordingly, if the presumption is true, information about adverse effects from the healthcare providers to patients and their parent(s) was in compliance with recommendations in the majority of cases. On the other hand, patients who were treated with SSRIs for depression, and patients with previous suicidality and outpatients were significantly more likely to receive information specifically about the risk of suicidality. Thus, for these patient groups, the clinicians find it important to emphasize the risk of suicidality as an adverse effect. The clinical guidelines deviate on how to handle SSRI-related suicidality when treating OCD and anxiety [11, 14–16]. Guidelines from The Danish Health Authority do not specify that patients who are treated with SSRIs for OCD or anxiety should be informed about suicidality on drug initiation. However, suicidality is not a symptom restricted to depression. In fact, mental illness of all diagnostic groups is a well-documented risk factor for suicidality [27]. Between 36% and 63% of adults with OCD experience suicidal ideation [28]. Less is known about suicidal ideation in children and adolescents with OCD, but in one study of 54 paediatric patients with OCD, 13% experienced suicidal ideation [28]. Risk factors for SSRI-related suicidality have not been fully investigated [29], and hence, specific clinical subgroups with heightened risk of suicidality during SSRI treatment have not been identified. Patients who had displayed suicidality prior to SSRI initiation were more likely to be informed about adverse effects and the risk of suicidality than patients with no prior suicidality. Previous suicidality is a known risk factor for suicidality and the finding may reflect that clinicians are alert of the risk of SSRI-related suicidality for patients at risk.

### Monitoring for suicidality

The majority (83.3%) of patients were monitored for suicidality after SSRI initiation and 67.7% of patients were monitored within 4–6 weeks after SSRI initiation, as specified by the clinical guidelines from The Danish Health Authority. Busch et al. [30] assessed changes in monitoring of patients treated with antidepressants after FDA warnings in the USA. Monitoring was defined as at least two outpatient visits within 30 days of initial drug treatment. In the study, 28–30% of the children were monitored after initial drug treatment. Comparatively, the patients in the present study were more intensely monitored.

Patients who were treated with SSRIs for depression were more likely to be monitored for suicidality than patients who were treated with SSRIs for other diagnostic indications. Since suicidal ideation and behaviour are typical clinical manifestations of depression, patients with depression or depressive symptoms are routinely monitored throughout their illness for suicidality in the clinic which might explain this difference. However, as noted above, symptoms of suicidality are not restricted to depressive disorders, and little is known about other risk factors for SSRI-related suicidality [27–29]. One well-established risk factor for suicide in general is previous suicidality. Patients with suicidality prior to SSRI initiation were more likely to be monitored for suicidality after SSRI initiation than patients who had not been suicidal before. This suggests heightened clinician alertness for these patients as well as common clinical practice of routine monitoring when there is a history of suicidality.

In the present study, younger age was a risk factor for failure to monitor for suicidality. This finding might reflect an assumption that suicidality is unlikely in younger children. Historically, children were believed not to be able to exhibit suicidality [31]. Today, the literature illustrates that children can commit suicide, attempt suicide, and have suicidal ideations as early as preschool age [31]. Meta-analyses evaluating SSRI-related suicidality have not separated data for children and adolescents. As a result, it is unknown if SSRI-related suicidality differs between children and adolescents. Government warnings as well as clinical guidelines do not clearly distinguish between monitoring children and adolescents during SSRI treatment.

SSRI initiation before 2013 was a risk factor for less monitoring. The observed difference implies a delay in the implementation of new knowledge, since the guidelines from The Danish Health Authority from 2007, 2012 and 2013 all emphasize monitoring for suicidality after SSRI initiation [11, 21].

Just more than half of the patients in the present study were monitored for suicidality at SSRI initiation. Registering baseline suicidality when initiating SSRI treatment is not specifically recommended in the clinical guidelines from The Danish Health Authority. On the other hand, it is good clinical practice to monitor symptoms that may later occur as side effects before initiating medical treatment [13].

### Non-pharmacological interventions

The greatest part of patients in this study received non-pharmacological interventions for a broad range of indications both prior to and in parallel with SSRI treatment. In this study, all but two inpatients received non-pharmacological interventions prior to, and all inpatients were treated with non-pharmacological interventions in parallel with SSRI treatment. Since hospitalization, both acute and planned, was registered as a non-pharmacological intervention, this finding is evident. There were no other significant predictors for not providing non-pharmacological interventions. Contrary to other diagnoses, for severe depression, combination treatment can be considered as first-line treatment according to recommendations [11]. Thus, patients with this diagnosis were expected to be less likely to receive non-pharmacological interventions alone prior to SSRI treatment, but no relationship between a diagnosis of severe depression and absence of non-pharmacological interventions prior to SSRI treatment was found.

### Study strengths and limitations

The strength of this study was the systematic extraction of data from digitalised prescription and medical record software for a large number of patients in a public hospital service in the most populous region of Denmark. The method of retrieving data from the clinical database ensured a maximal entry of eligible participants. Data were extracted on patient level, which allowed for detailed clinical data on treatment with SSRIs. By extracting data directly from the digitalised software, recall bias and non-response were avoided. Our findings are by large generalizable to the whole population of SSRI-treated children in Denmark, due to the Danish health authorities limiting prescriptions of any psychopharmacological drug to be handled by specialists in child and adolescent psychiatry, i.e., general practitioners must refer children and adolescents in need for psychiatric drug treatment to the specialist treatment. The majority of these specialists are affiliated with the hospital CAMHS clinics. In 2016, 65.7% of first prescriptions for antidepressants for children and adolescents were prescribed by doctors affiliated with hospital clinics, and 80.9% of children and adolescents with a psychiatric diagnosis had at least one hospital contact in 2016 [32]. Furthermore, the CAMHS in Copenhagen is the largest in the country, and all other CAMHS have similar diagnostic patient distributions, and follow the same national guidelines. It cannot, however, be concluded that all CAMHS have exactly the same adherence to national guidelines as the CAMHS Copenhagen.

The main limitations of this study are information bias and the risk of misclassification. Healthcare providers in Denmark are liable [33] to record information about patients’ conditions, treatment, and information given from healthcare providers to the patient. Nevertheless, information bias might occur, because the medical record is not a meticulous report of the consultation and because of limited information on the use of non-pharmacological interventions outside of CAMHS (i.e., the social services, private sector, and school counsellors). Documentation in the medical journal is a limited way to measure information about adverse effects, since it is unknown to what degree the information is understood by patient or their parent(s). In this study, the focus is rather on the clinicians’ awareness to recommendations.

To account for the risk of misclassification, the extraction of data was restricted to only two researchers, and doubts were solved in a standardized way. As an example, if in doubt whether an event was in the category “supportive consultations” or “psychotherapy”, the event was systematically classified as “supportive consultations”.

It could have been interesting to collect data on the type of treatment facility within the CAMHS and which healthcare providers treated the patients. Specific healthcare providers might be less likely to comply with clinical guidelines than others, thus interfering with the results of this study.

## Conclusion

The present study aimed to determine whether daily clinical practice complied with specific recommendations using data from digitalized prescription software and digitalized medical records. The study method emphasizes the feasibility of monitoring clinical practice by digitalized data.

The study found some deviances from recommendations concerning the management of suicidality when treating youth with SSRIs. Of important clinical relevance was the observation that patients of younger age and/or with diagnostic indications for SSRI treatment other than depression were less likely to be managed according to recommendations. Patients at risk, i.e., with previous suicidality, were more likely to be managed according to recommendations. Clinicians were less likely to inform about adverse effects and monitor for suicidality when SSRI initiation was before 2013. This stresses the responsibility of authorities in issuing prompt recommendations as early as possible, as it has potential of affecting clinical practice.

These findings have implications for improving clinical practice, i.e., it might be considered that clinical guidelines highlight the need for clinicians to increase awareness on suicidality when treating youth with SSRIs regardless of patient age and diagnosis. Furthermore, our findings emphasize the need for future research in SSRI-related suicidality to identify subgroups at risk and specific time-periods for increased risk of suicidality during SSRI treatment. Until these issues are clarified, assessment and pre-consent information of suicidality are necessary as a precautionary measure for all children and adolescents treated with SSRIs. Clinicians should be cautious when treating patients at younger age and patients with another indication for SSRI treatment than depression, since these subgroups are less likely to be informed about and monitored for suicidality.
